# Characterization of differences in seed endophytic microbiome in conventional and organic rice by amplicon-based sequencing and culturing methods

**DOI:** 10.1128/spectrum.03662-23

**Published:** 2024-08-13

**Authors:** Sabin Khanal, Muhammad Imran, Xin-Gen Zhou, Sanjay Antony-Babu

**Affiliations:** 1 Texas A&M AgriLife Research Center, Beaumont, Texas, USA; 2 Department of Plant Pathology, University of Faisalabad, Faisalabad, Pakistan; 3 Department of Plant Pathology and Microbiology, Texas A&M University, College Station, Texas, USA; University of Arkansas for Medical Sciences, Little Rock, Arkansas, USA

**Keywords:** organic farming, conventional farming, rice seed microbiome, biocontrol

## Abstract

The seed serves as the primary source for establishing microbial populations in plants across subsequent generations, influencing plant growth and overall health. Cropping conditions, especially farming practices, can influence the composition and functionality of the seed microbiome. Very little is known about the differences in seed microbiome between organic and conventional production systems. In this study, we characterized the endophytic microbial populations in seeds of rice grown under organic and conventional management practices through culture-dependent and -independent analyses. The V4 region of 16S rRNA was used for bacterial taxa identification, and the ITS1 region was used for the identification of fungal taxa. Our results revealed significantly higher Shannon and Simpson indices for bacterial diversity in the conventional farming system, whereas the fungal diversity was higher for observed, Shannon, and Simpson indices in the organic farming system. The cultivable endophytic bacteria were isolated and identified using the full-length 16S rRNA gene. There was no difference in culturable endophytic bacterial isolates in rice seeds grown under both conventional and organic farming systems. Among 33 unique isolates tested *in vitro*, three bacteria—*Bacillus* sp. ST24, *Burkholderia* sp. OR5, and *Pantoea* sp. ST25—showed antagonistic activities against *Marasmius graminum, Rhizoctonia solani* AG4, and *R. solani* AG11, the fungal pathogens causing seedling blight in rice.

**IMPORTANCE:**

In this paper, we studied the differences in the endophytic microbial composition of rice seeds grown in conventional and organic farming systems. Our results demonstrate a greater bacterial diversity in conventional farming, while organic farming showcases a higher fungal diversity. Additionally, our research reveals the ability of seed bacterial endophytes to inhibit the growth of three fungal pathogens responsible for causing seedling blight in rice. This study provides valuable insights into the potential use of beneficial seed microbial endophytes for developing a novel microbiome-based strategy in the management of rice diseases. Such an approach has the potential to enhance overall plant health and improve crop productivity.

## INTRODUCTION

Plant-associated microbial communities affect various plant traits (1). The plant microbiota can be acquired through either vertical transmission from the parent or horizontal transmission from the environment (2). While the seed microbiota act as a primary influencer for the overall plant microbiome (and hence the plant health), they can also play a direct role in seed germination and inducing tolerance to biotic and abiotic stresses (3, 4). Multiple contributing factors can affect plant microbial community structures, such as environmental perturbation, soil composition and microbes, plant genotype, etc. (5, 6). Among the environmental factors, farming practices play an important role in microbial community diversity (7). The seed microbiomes are estimated to have more than 9,000 different microbial taxa, which can engage in synergistic, commensal, and potentially pathogenic interactions with their host plants (8). Bacterial endophytes are among the most common microbes, with *Actinobacteria, Bacteroidetes, Firmicutes,* and *Proteobacteria* being the representative phyla (9–12). Similarly, several fungal classes such as Dothideomycetes*,* Eurotiomycetes*,* Leotiomycetes*,* Sordariomycetes*,* and Tremellomycetes are also reported as endophytic fungal populations in seeds (13). In the study presented here, we investigated the seed microbiota of rice. Previous studies examining the influence of conventional and organic farming systems have primarily focused on microbial communities in the rhizosphere soil (14–17). These studies provide evidence of greater species abundance and higher diversity within the rhizosphere of organic production systems. Some studies have also shown the ability of seed microbial populations to protect seedlings from various soilborne pathogens (18, 19). Especially, seed endophytes with antifungal properties hold promise as potential biocontrol agents in agricultural settings (13, 20).

The demand for organic rice has led to an almost six-fold increase in organic rice production in the US since 1995. Texas and California, together, account for 76% of this increase and stand as the largest organic rice-producing states. With increased production, it is critical to design effective management strategies against crop loss due to diseases, harmful insects, and weeds. However, organic rice growers currently lack effective management tools for these rice pests (21). The identification and development of effective biocontrol agents can offer rice farmers such a tool for disease management, especially considering that the use of synthetic fungicides is prohibited in organic production systems. In addition, conventional rice growers are interested in adopting integrated disease management practices for rice production. The integrated use of biocontrol agents with conventional synthetic products can improve control efficacy, increase the longevity of the chemicals, and decrease the chemical load into the environment (22, 23). Several bacterial taxa are known to have antagonistic properties against fungal pathogens and have the potential as disease biocontrol agents (24, 25). Many bacterial taxa are known to possess biocontrol properties against fungal pathogens of rice and other crops, especially members of the genera *Bacillus*, *Pantoea*, *Pseudomonas*, and *Streptomyces* (23, 26). These bacterial species target various fungal pathogens ranging from those found in the soil to those affecting foliage (27–32).

Seedling blight is an important disease in rice in the southern US, causing irregular, thin stands and weakened plants, ultimately resulting in significant stand loss and yield reduction (33). Dry seedling is the predominant method for rice cultivation in Texas and other southern states. Early plantings are widely practiced in Texas and Louisiana, allowing for the production of a second crop (ratoon) while reducing the likelihood of heat stress and late-season disease occurrence. In such production conditions, seedling blight has arisen as the primary factor contributing to stand loss. This is primarily due to the onset of cold soil temperatures during early stages, which facilitates the development of seedling blight (34, 35). Seedling blight causes pre- and post-emergence damping off of rice plants. Although some blighted seedlings survive, they still suffer from poor vigor and have stunted growth. According to the most recent seedling disease surveys, *Rhizoctonia solani* AG11 is the most abundant fungal pathogen that causes seedling blight in the southern US (34). *R. solani* AG4 and *Marasminus graminum,* the two new fungal pathogens, also cause the seedling blight disease (34, 36, 37). Currently, farmers heavily rely on fungicide seed treatment for the control of these seedling blight (33, 38).

The objective of this study was to examine the impact of farming practices, specifically conventional and organic farming, on seed microbial composition and to identify the potential major microbial components involved. Our goal is to lay the foundation for larger studies across different geographical regions worldwide. To the best of our knowledge, this is the first study examining the diversity of seed endophytic microbial communities in the spermosphere of conventionally and organically grown rice. Additionally, we aimed to identify naturally inhabiting microorganisms that held potential for managing seedling blight in rice. Our results indicate that the diversity of endophytic bacterial communities in rice seeds was significantly higher in the conventional farming system compared to the organic system. Conversely, the diversity of endophytic fungal communities was greater in organically grown rice seeds. Furthermore, we observed that seed endophytic bacteria exhibited antagonistic properties against three seedling blight-causing pathogens in rice: *R. solani* AG11, *R. solani* AG4, and *M. graminum*.

## RESULTS

### Microbial communities associated with rice seed endosphere

**Fig 1:**
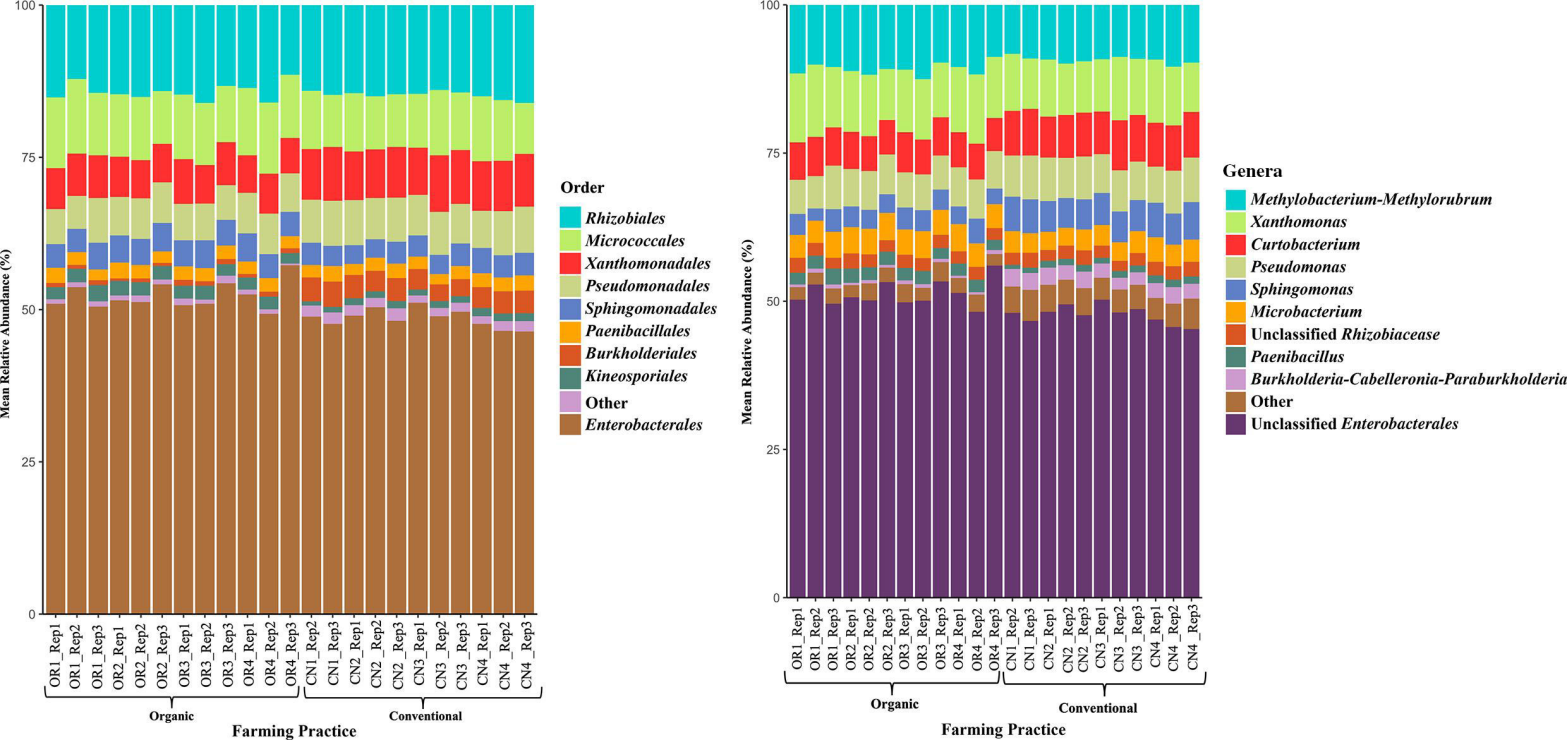
Relative abundance of seed endophytic bacterial populations in rice grown in organic and conventional farming systems based on 16S rRNA V4 region amplicon sequencing. Bacterial taxa with less than 2% relative abundance are placed in the “other” category. OR represents the seed samples collected from organic rice, whereas CN represents the seed samples from conventional rice.

A combined total of 1,474,695 sequences were obtained for 16S rRNA V4 region sequencing, while 1,132,133 sequences were acquired for internal transcribed space (ITS) amplicon sequencing. One sample from 16S rRNA sequences was excluded from analysis due to extremely low-quality sequences. Analysis of 16S rRNA amplicons identified a total of 78 operational taxonomic units (OTUs) after removal of single digit (<10) OTUs. The relative abundance of bacterial orders within the core community across all the seed samples was Enterobacterales: 46.38%–57.39%, followed by Rhizobiales:11.48%–16.08%, Micrococcales*:* 8.35%–12.19%, Xanthomonadales: 5.85%–9.25%, Pseudomonadales: 5.45%–7.54%, Sphingomonadales: 3.05%–4.63%, Paenibacillaceae: 1.71%–2.60%, Burkholderiales: 0.44%–4.17%, Kineosporiales: 0.69%–2.71%, and other orders with less than 2% on all the samples were placed in “others” category (Fig. 1; Table S1). These 9 orders were classified into 11 different genera, where unclassified Enterobacterales dominated the classification among both treatments.

**Fig 2:**
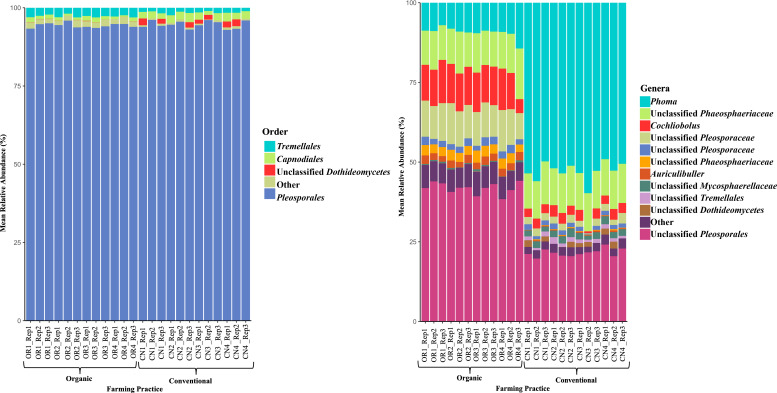
Relative abundance of seed endophytic fungal populations in rice grown in organic and conventional farming systems based on ITS amplicon sequencing. Fungal taxa with less than 2% relative abundance are placed in the “other” category. OR represents the seed samples collected from organic rice, whereas CN represents the seed samples from conventional rice.

Similarly, ITS amplicons sequencing analysis identified a total of 54 OTUs after removal of single digit (<10) OTUs. The Pleosporales were the dominant fungal order, accounting for >90% relative abundance in all samples (Fig. 2; Table S2). When classifying the OTUs at the genus level, we observed numerical differences in the relative abundance of *Phoma,* unclassified Pleosporales*,* and *Cochliobolus* between the farming practice treatments. In conventional rice seeds, the fungal genus *Phoma* was the dominant fungal genera, ranging from 49.08% to 55.98%, while in organic rice seeds, it ranged from 9.18% to 14.34%. In organic rice seeds, the unclassified Pleosporales dominated the classification, ranging from 38.33% to 41.01%, while in conventional rice seeds, it ranged from 19.7% to 24.09%. In addition, organic rice seeds exhibited a higher abundance of fungal genus *Cochliobolus* compared to conventional rice seeds, ranging from 4.36% to 13.04% and 0.57% to 3.60%, respectively.

### Difference in microbial diversity between conventional and organic rice seeds

**Fig 3:**
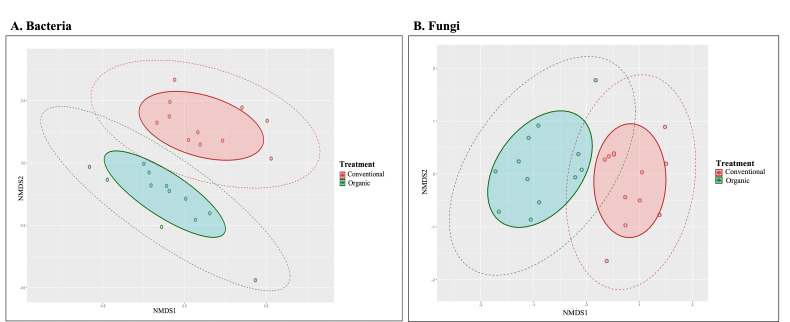
Nonmetric multidimensional scaling ordination of variation in bacterial (**A**) and fungal (**B**) community structure (Bray-Curtis distance) in the endophytic microbial populations of rice seeds grown in organic and conventional farming systems. PERMANOVA based on Bray-Curtis distance, bacteria = 0.001 (*R*^2^ = 0.719), fungi = 0.001 (*R*^2^ = 0.964). Shaded area represents 75% of the core composition, whereas dashed line represents 95% of the composition.

The nonmetric multidimensional scaling (NMDS) ordination of variations in bacterial and fungal communities showed strong clustering of endophytic microbial assemblage among the seeds obtained from conventional and organic farming systems (Fig. 3). The observed difference between the two farming systems based on the Bray-Curtis distance was significant. The results of permutational multivariate analysis of variance (PERMANOVA) conducted on the Bray-Curtis distance showed that the difference in farming system explained 71.9% (*P* < 0.001) of the variation in the bacterial community, whereas in the fungal community, the analysis explained 96.4% (*P* < 0.001) of the variation could be attributed to the difference between the two farming systems.

**Fig 4:**
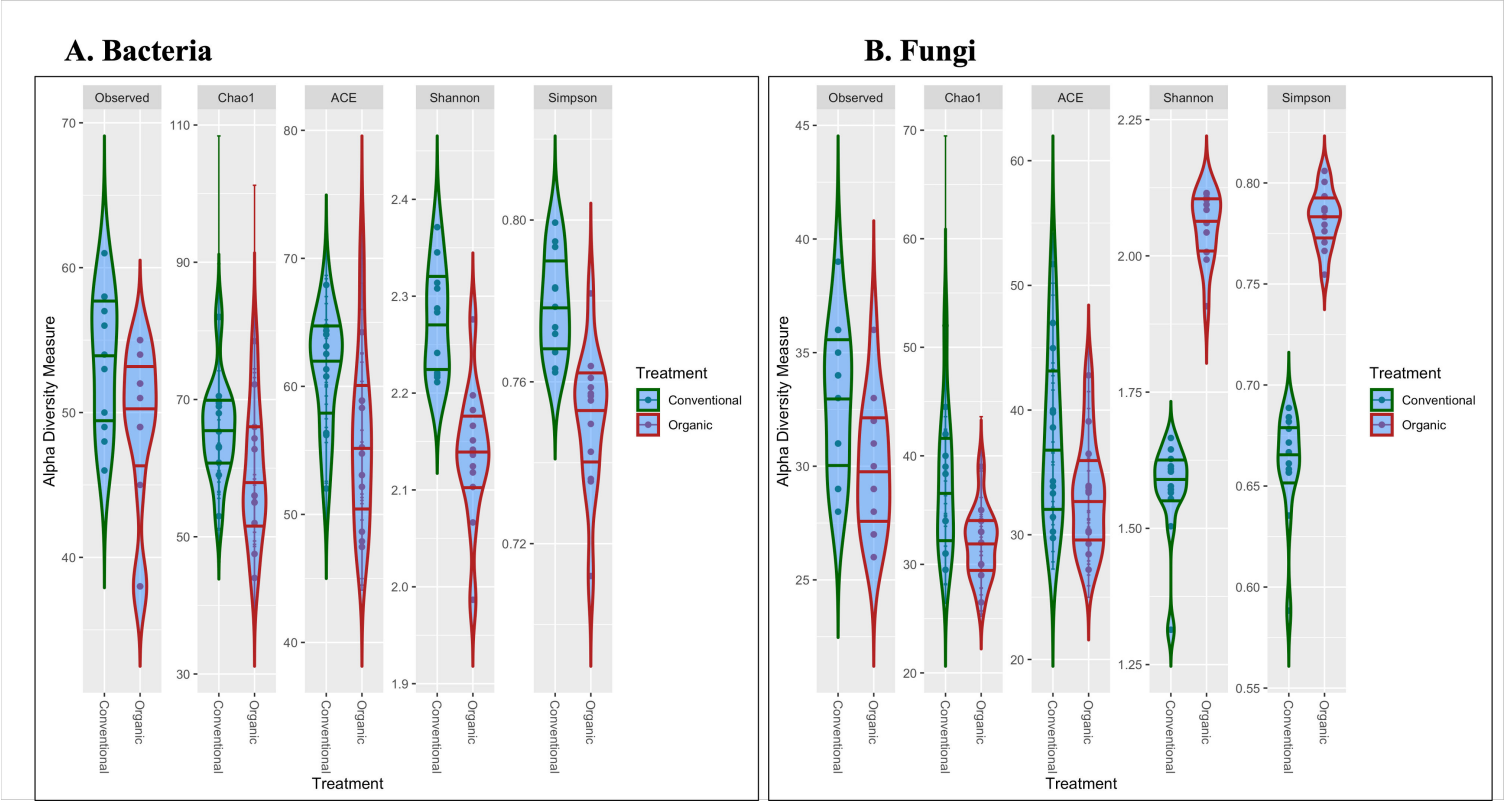
Diversity indices (observed, chao, ACE, Shannon, and Simpson) of bacterial (**A**) and fungal (**B**) communities in endophytic microbial populations in rice seeds grown in organic and conventional farming systems. Each point in the violin-blot indicates the individual sample. (A) Observed (*U* = 95, df = 1, *P* = 0.07831), Chao1 (*U* = 94, df = 1, *P* = 0.09039), ACE (*U* = 99, df = 1, *P* < 0.05), Shannon (*U* = 127, df = 1, *P* < 0.0001), and Simpson (*U* = 8, df = 1, *P* < 0.0001). (B) Observed (*U* = 107, df = 1, *P* < 0.05), Chao1 (*U* = 112, df = 1, *P* < 0.02205), ACE (*U* = 95, df = 1, *P* = 0.1978), Shannon (*U* = 0, df = 1, *P* < 0.0001), and Simpson (*U* = 144, df = 1, *P* < 0.0001).

Similarly, an analysis of alpha diversity measured was performed on observed, Shannon, and Simpson indices. We used Mann-Whitney *U* test to analyze the alpha diversity measures. Our analysis on the bacterial community (Fig. 4A) showed among community richness, only the ACE estimator ACE (*U* = 99, df = 1, *P* < 0.05) was significantly different, whereas there was no significant difference in observed/sobs (*U* = 95, df = 1, *P* = 0.07831) and other community richness indices Chao1 (*U* = 94, df = 1, *P* = 0.09039) between bacterial communities in the conventional and organic rice seeds. In contrast, both indices, Shannon (*U* = 127, df = 1, *P* < 0.0001) and Simpson (*U* = 8, df = 1, *P* < 0.0001), were significantly higher in conventional rice seeds in comparison to organic rice seeds. Analysis of diversity indices on fungal community (Fig. 4B) showed significantly higher diversity on community richness indices, observed/sobs (*U* = 107, df = 1, *P* < 0.05), and community richness indices in Chao1 (*U* = 112, df = 1, *P* < 0.02205), as well as both Shannon (*U* = 0, df = 1, *P* < 0.0001) and Simpson (*U* = 144, df = 1, *P* < 0.0001) indices on organic rice seeds in comparison to conventional rice seeds. Only one community richness index, ACE (*U* = 95, df = 1, *P* = 0.1978), showed no significant differences between fungal communities in organic rice seeds compared to conventional rice seeds.

### Drivers of variation in community composition and diversity

**Fig 5:**
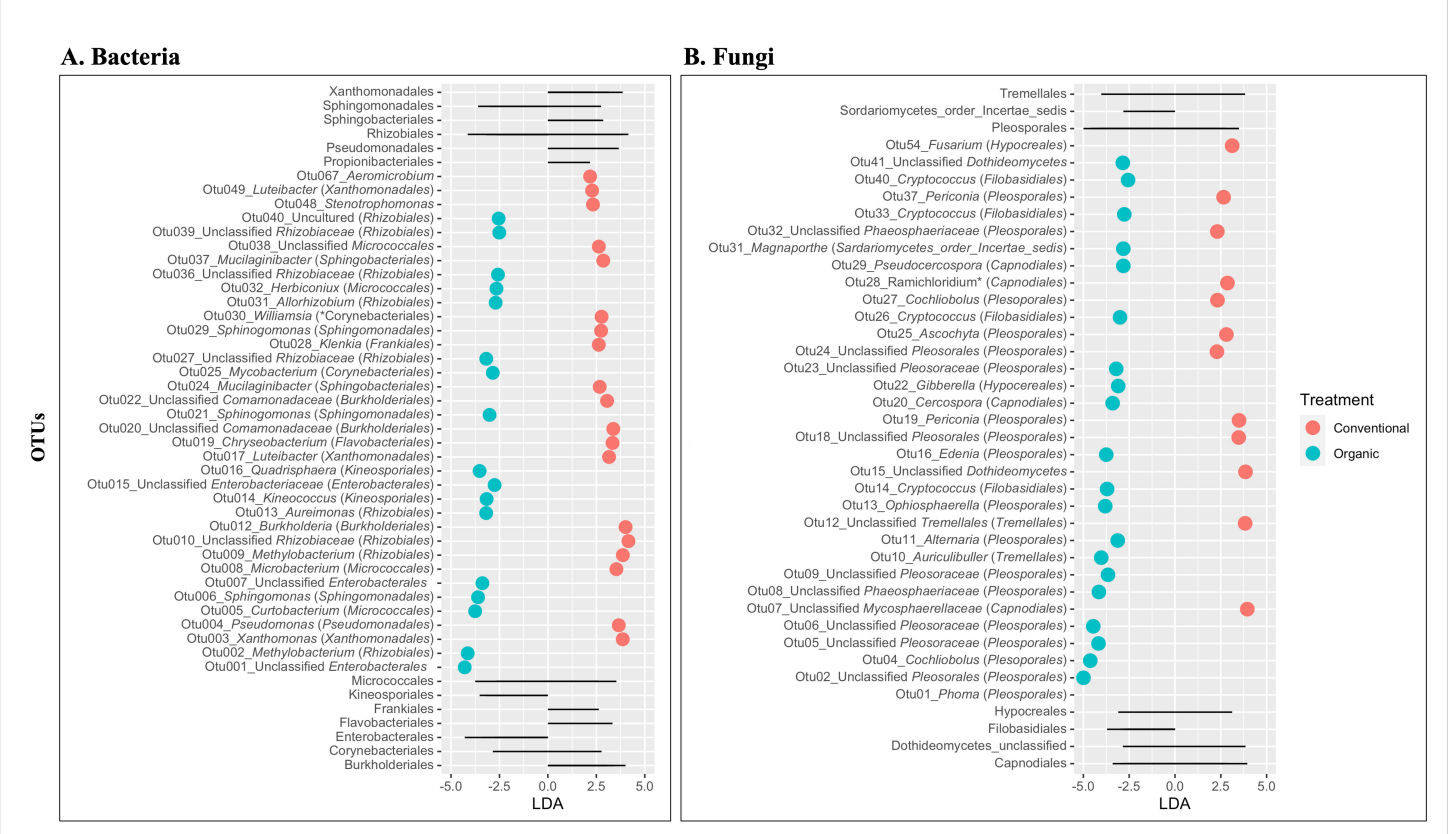
Least discriminatory analysis effect size on bacterial (**A**) and fungal (**B**) communities of the endophytic microbial populations in rice seeds grown in conventional and organic farming systems. Significance was determined using default parameters (Kruskal Wallis test *P* < 0.05 and LDA score >2). Each point represents the magnitude of effect size by specific OTUs in individual treatments. Gray lines on top and bottom indicate the orders of OTUs that are differentially abundant in both bacterial and fungal communities.

The linear discriminatory analysis (LDA) effect size (Lefse) was conducted on both bacterial (Fig. 5A) and fungal (Fig. 5B) communities. Our analysis revealed the differentially abundant OTUs between the conventional and organic rice seeds. There were 36 bacterial OTUs differentially abundant in bacterial taxa, with 19 OTUs differentially abundant in conventional rice seeds and the 17 OTUs in organic rice seeds (Fig. 5A). In addition, we also analyzed the indicator OTUs, where we defined indicators as specific OTUs present across all the samples of one single treatment. Our analysis revealed 12 indicator OTUs in the rice seeds from conventional farming system: Otu009 (genus: *Methylobacterium*, order: Rhizobiales), Otu017 (genus: *Luteibacter*, order: Xanthomonadales), Otu022 (genus: unclassified *Comamonadaceae*, order: Burkholderiales), Otu024 (genus: *Mucilaginibacter*, order: Sphingobacteriales), Otu029 (genus: *Sphingomonas*, order: Sphingomonadales), Otu030 (genus: *Williamsia*, order: Corynebacteriales), Otu038 (genus: unclassified *Microbacteriaceae*, order: Micrococcales), Otu048 (genus: *Stenotrophomonas*, order: Xanthomonadales), Otu052 (unclassified *Saccharimonadales*), Otu058 (genus: *Bosea*, order: Rhizobiales), Otu062 (genus: *Novosphingobium*, order: Sphingomonadales), and Otu067 (genus: *Aeromicrobium*, order: Propionibacteriales). Nine out of 12 OTUs overlapped with differentially abundant OTUs in conventional rice (Fig. 5A). We did not find any indicator OTUs in rice seeds from the organic farming system.

Similarly, Lefse analysis on the fungal community (Fig. 5B) revealed 34 differentially abundant OTUs. Among these, 13 OTUs were differentially abundant in rice seeds from the conventional farming system, while 21 OTUs were in rice seeds from the organic farming system. Our indicator analysis revealed eight indicator OTUs in rice seeds from the conventional farming system: Otu01 (genus: *Phoma*, order: Pleosporales), Otu07 (genus: unclassified *Mycosphaerellaceae*, order: Capnodiales), Otu015 (unclassified *Dothideomycetes*), Otu018 (unclassified Pleosporiales), Otu028 (genus: *Ramichloridium*, order: Capnodiales), Otu032 (unclassified *Phaeosphaeriacea,* order: Pleosporales), Otu037 (genus: *Periconia*, order: Pleosporales), and Otu054 (genus: *Fusarium,* order: Hypocreales). All eight indicator fungal OTUs overlapped with 13 OTUs differentially abundant in the conventional farming systems (Fig. 5B).

### Culture-dependent bacterial diversity

Bacterial endophytes were isolated from rice seeds grown in conventional and organic farming systems. Titer counts of seed endophytic bacteria were 6 × 10^8^ and 7 × 10^8^ cfu/mL for conventional and organic rice seeds, respectively. A total of 39 and 46 bacterial endophytes were isolated and purified from conventional and organic rice seeds, respectively (Table 1). All 85 bacterial isolates were subjected to near full-length 16S rRNA sequencing for identification. Bacteria belonging to genera *Pantoea* and *Pseudomonas* dominated the seed endosphere in both farming systems. *Pantoea* isolates accounted for 41% of the total bacterial seed endophytic community in conventional rice seeds and 33% in organic rice seeds. Similarly, *Pseudomonas* spp. accounted for 20% and 21% of the total seed endophytic bacteria in conventional and organic rice seeds, respectively. Among the rare isolates, *Curtobacterium* spp. accounted for 5% of seed endophytic bacterial isolates in conventional rice seeds, whereas they covered 13% of seed endophytic bacterial isolates in organic rice seeds. Furthermore, two isolates of *Bacillus* spp. were identified in conventional rice seeds, while one isolate each of *Paenibacillus* spp. and *Chryseobacterium* spp. was found in organic rice seeds (Table 1).

**TABLE 1:** Identification of culturable seed endophytic bacteria isolated from rice seeds grown in conventional and organic farming systems

Genera^*a*^	Species^*b*^	Conventional rice	Organic rice
*Arthrobacter*	*A. globiformis*	–^*c*^	1
	*Arthrobacter* sp.^*d*^	1	–
*Bacillus*	*B. albus*	1	–
	*Bacillus* sp.	1	–
*Burkholderia*	*B. ubonensis*	–	3
	*Burkholderia* spp.^*e*^	3	–
*Chryseobacterium*	*C. endophyticum*	–	1
*Curtobacterium*	*C. albidium*	–	6
	*C. leutum*	1	–
	*Curtobacterium* spp.	1	–
*Paenibacillus*	*P. taichungensis*	–	1
*Pantoea*	*P. eucrina*	–	1
	*P. dispersa*	–	3
	*P. vagans*	2	3
	*Pantoea* spp.	14	8
*Pseudomonas*	*P. oryzihabitans*	1	8
	*Pseudomonas* spp.	7	2
*Sphingomonas*	*S. sanguinis*	1	3
*Xanthomonas*	*X. sontii*	1	6
	*Xanthomonas* spp.	5	–
	Total	39	46

^*a*^ Genera of bacteria identified by blast.

^*b*^ Taxon of genera identified by blast, isolates with >97% identity were grouped in same taxa.

^*c*^ No isolates available in this category.

^*d*^ Species delineation of the bacterial genera was not clear.

^*e*^ Multiple taxa of same genera.

### Antagonistic activity of bacterial endophytes against seedling blight pathogens

From the endophytic bacteria isolated from both conventional and organic rice seeds, 31 representative isolates were randomly selected based on their 16S rRNA sequence differences. Isolates with >97% sequence similarities were grouped together as putative same “species,” and a representative isolate was selected from each group for further study. Out of the 31 isolates selected, 11 were derived from organic rice seeds, while the remaining 20 were isolated from conventional rice seeds. *In vitro* assays were conducted with the 31 different bacterial isolates to evaluate their antagonistic activity against three rice seedling blight pathogens: *M. graminum, R. solani* AG4, and *R. solani* AG11. Three isolates, *Bacillus* sp. ST24, *Burkholderia* sp. OR5, and *Pantoea* sp. ST25, exhibited strongest antagonistic properties against the three seedling blight pathogens. (Table 2; Fig. S1). These three bacterial isolates were able to inhibit more than 50% of the mycelium growth in all three seedling blight pathogens. On the contrary, two *Burkholderia* isolates, ST35 and ST43, showed radial enhancement in *R. solani* AG4 (Table 2). Additionally, the phylogenetic analysis was performed on three bacterial species, *Bacillus*, *Burkholderia*, and *Pantoea,* using 16S rRNA of each sequences available for each species in databases (https://www.ncbi.nlm.nih.gov/nuccore) and RDP (https://sourceforge.net/projects/rdp-classifier/). The phylogenetic analyses conducted for all three bacteria species resulted in their separate classification within distinct clades (Fig. S2).

**TABLE 2:** Identification of seed endophytic bacteria and their *in vitro* antagonistic activities against the three rice seedling blight pathogens, *Rhizoctonia solani* AG4, *R. solani* AG11, and *Marasmius graminum*

S. no.	Genera^*a*^	Species^*b*^	Isolate^*c*^	Mycelium growth inhibition (%)^*f*^		
				*R. solani* AG4	*R. solani* AG11	*M. graminum*
1	*Arthrobacter*	*A. globiformis*	OR6	0.7 ± 0.5 jklv	0.6 ± 3.1 h	20 ± 4.5 gh
2	*Arthrobacter*	*Arthrobacter* sp.^*d*^	ST16	4.3 ± 0.7 ijk	27.2 ± 3.9 def	18.3 ± 1.5 gh
3	*Bacillus*	*B. albus*	ST23	28.6 ± 0.7 efgh	26.5 ± 8.8 def	38 ± 3.1 f
4	*Bacillus*	*Bacillus* sp.	ST24	66 ± 1.9 b	71.9 ± 0.6 a	57.5 ± 0.3 d
5	*Burkholderia*	*B. ubonensis*	OR5	66 ± 1.3 b	70.9 ± 6.2 a	79.3 ± 0.9 a
6	*Burkholderia*	*Burkholderia* sp.	ST35	–^*e*^	42.8 ± 11.7 bc	17.5 ± 3.6 gh
7	*Burkholderia*	*Burkholderia* sp.	ST43	–	13.4 ± 7.7 fgh	9.4 ± 2.2 ghij
8	*Chryseobacterium*	*C. endophyticum*	OR23	10.4 ± 4 i	20.3 ± 7.2 efg	18 ± 1.4 gh
9	*Curtobacterium*	*C. albidum*	OR1	20.7 ± 2.5 h	31.4 ± 1.4 cde	78.3 ± 2.5 ab
10	*Curtobacterium*	*Curtobacterium* sp.	ST9	27.5 ± 0.9 efgh	25.5 ± 11.6 def	16.5 ± 0.5 gh
11	*Curtobacterium*	*Curtobacterium* sp.	ST18	26.8 ± 2.3 efgh	30.7 ± 4.4 cde	19.8 ± 1.5 ghi
12	*Paenibacillus*	*P. taichungensis*	OR41	35.3 ± 7.7 de	26.9 ± 1.3 def	11.6 ± 0.5 ghij
13	*Pantoea*	*P. vagans*	OR2	33.6 ± 1.9 defg	22.4 ± 5.7 defg	21.7 ± 2.2 g
14	*Pantoea*	*P. eucrina*	OR27	49.6 ± 1.6 c	25.8 ± 1.3 def	64.2 ± 4.4 cd
15	*Pantoea*	*Pantoea* sp.	OR39	33.2 ± 0.1 defg	21.3 ± 3.7 efg	6.9 ± 3.8 hij
16	*Pantoea*	*Pantoea* sp.	ST14	26.1 ± 1.1 fgh	18.2 ± 2.4 efg	7.2 ± 4 hij
17	*Pantoea*	*Pantoea* sp.	ST25	66.4 ± 1.8 a	67.4 ± 5.3 a	7.2 ± 4 hij
18	*Pantoea*	*Pantoea* sp.	ST30	34.6 ± 3.1 def	47.5 ± 6.3 b	54.1 ± 2.7 de
19	*Pantoea*	*P. dispersa*	ST26	35 ± 1.2 de	47.7 ± 6.3 b	6.9 ± 3.8 hij
20	*Pantoea*	*Pantoea* sp.	ST33	27.1 ± 1 efgh	29.3 ± 2.2 cde	6.9 ± 3.8 hij
21	*Pseudomonas*	*P. oryzihabitans*	OR4	34.3 ± 5.3 def	27.9 ± 3.3 def	3.2 ± 3.9 ij
22	*Pseudomonas*	*Pseudomonas* sp.	ST5	26.8 ± 1.1 efgh	32.4 ± 4.7 cde	11.8 ± 1.1 ghij
23	*Pseudomonas*	*Pseudomonas* sp.	ST11	29.3 ± 4.6 efgh	29.7 ± 0.9 cde	57.8 ± 2.2 d
24	*Pseudomonas*	*Pseudomonas* sp.	ST17	28.6 ± 7.6 efgh	17.9 ± 6.3 efg	76.5 ± 0.3 abc
25	*Pseudomonas*	*Pseudomonas* sp.	ST27	32.9 ± 5.2 def	31.4 ± 2.6 cde	64.9 ± 7.1 bcd
26	*Pseudomonas*	*Pseudomonas* sp.	ST20	40 ± 1.9 d	32.4 ± 1.6 cde	64.9 ± 1.8 bcd
27	*Pseudomonas*	*Pseudomonas* sp.	ST38	28.2 ± 0.7 efgh	28.6 ± 1.3 cde	62.7 ± 1.7 d
28	*Sphingomonas*	*S. sanguinis*	OR34	6.1 ± 1.6 ij	22 ± 6.3 defg	11.9 ± 1.1 ghij
29	*Xanthomonas*	*X. sontii*	OR7	25.4 ± 1.3 gh	36.2 ± 4.7 bcd	11.9 ± 1.1 ghij
30	*Xanthomonas*	*Xanthomonas* sp.	ST21	3.2 ± 0.7 ijk	9.2 ± 2.8 gh	7.2 ± 4 hij
31	*Xanthomonas*	*Xanthomonas* sp.	ST28	10.4 ± 0.7 i	2.3 ± 2.1 h	0 ± 1.1 j

^*a*^ Genera of bacterial isolate.

^*b*^ Operational taxon of the bacterial isolate, isolates with >97% identity were grouped into same taxa.

^*c*^ OR indicates isolates recovered from organic rice, whereas ST indicates isolates from conventional rice.

^*d*^ Species delineation of the bacterial genera was not clear.

^*e*^ Two *Burkholderia* spp. isolates ST35 and ST43 were able to enhance the radial growth of *R. solani* AG4 by 7.85% and 2.85%, respectively, *in vitro*.

^*f*^ Means in each column followed by the same letter are not significantly different (*P <* 0.05) based on Fisher’s protected LSD test. Values indicated the mean inhibition percentage ± standard error.

## DISCUSSION

The plant endophytes residing within seeds (spermosphere) hold a significant role in the plant’s life cycle. They contribute to the maintenance of microbial populations across successive plant generations, making them integral to the plant’s life cycle (1, 2, 5). In addition to providing beneficial endosymbionts to the offspring, they also actively contribute to seed conservation, facilitate seed germination in soil, and enhance plant growth and defense mechanisms (1, 39–41). Horizontal transmission, alongside vertical transmission, contributes to the overall composition of the seed microbiota (1, 40). Previous studies have shown varying effects of farming practices on soil and root microbial communities in different cropping systems (12, 14, 15, 42–46). In this study, we employed both culture-independent and culture-dependent approaches to evaluate the endophytic microbial communities within rice seeds produced under conventional and organic farming systems.

The results of the current study demonstrate, for the first time, the differences in seed endophytic microbial communities between rice grown in conventional and organic farming systems. Eyre et al. (4) identified and characterized the core rice seed microbiome. However, their results are limited to conventional rice farming systems since their research was based on rice seeds harvested from rice crops grown under conventional management. In this study, we compared the microbial composition of rice seeds between conventional and organic production systems. Although the observed endophytic bacterial communities did not differ significantly between the two farming systems evaluated, alpha diversity measures revealed higher diversity and evenness of bacterial communities in the conventional farming system. Bacterial communities in seeds are highly variable and influenced not only by agricultural practices but also by host plant species (47). Therefore, further investigations are needed to gain a deeper understanding of whether the observed diversity between organic and conventional rice remains consistent across different genotypes or is restricted to specific ones. From the current study, *Proteobacteria, Actinobacteriota,* and *Bacteriota* were the most abundant phyla, consistent with the rice seed core microbiome reported previously (4). Additionally, in our study, the most prevalent genus observed was the unclassified *Enterobacterales.* However, these findings do not reveal a distinct pattern between the two farming systems. Some genera such as *Curtobacterium* and *Methylobacterium* were observed consistently in rice seeds grown under organic management, while *Burkholderia* was observed at a higher abundance in rice seeds from the conventional management system. Of these taxa, it is noteworthy that methylotrophic bacteria (such as *Methylobacterium)* are well known for their role as growth promoters in various host plants (48). All these observed taxa have been associated with the rice seed microbiome reported previously (4). Furthermore, these bacterial taxa have also been detected in soil microbiomes of both conventional and organic farming systems across a range of crops, including wheat, barley, maize, and other nonrice crops (15). Nonetheless, the transfer of soil microbiota to seed microbiota does not always occur directly, as various factors are involved during the establishment of seed microbial populations (1, 40).

Among the endophytic fungal communities, the ITS-region-based amplicon sequence data reveal a higher diversity of fungal communities in rice seeds grown under the organic farming system. The higher fungal diversity in the organic system over the conventional system can be attributed to the impacts of synthesized chemicals, especially fungicides, used in the conventional farming system. No synthesized chemicals were used in the organic farming systems, whereas various synthesized chemicals such as fungicides, insecticides, and herbicides were applied in the conventional farming system. It is well known that pesticides, especially fungicides, can selectively inhibit or eliminate certain groups of fungal and other microbial populations (15, 49). In the current study, *Ascomycota* and *Basidiomycota* were the only two phyla observed in rice seeds, consistent with the results of the rice core microbiome study reported previously (4). In contrast to the endophytic bacterial communities, we observed a clear taxonomic pattern within the fungal communities. Approximately 50% of the total genera found in rice seeds cultivated under conventional farming practices consisted of *Phoma* spp. These *Phoma* spp. are widely distributed and known as phytopathogens, sometimes associated with food contaminants with clinical significance, especially in immunocompromised individuals (50). Among the fungal communities in rice seeds grown in the organic farming system, unclassified *Pleosporales* dominated the fungal taxa. We also observed a higher abundance of *Cochliobolus* spp. in rice seeds grown in the organic farming system. Certain strains belonging to *Cochliobolus,* such as *Cochliobolus miyabeanus* (*Bipolaris oryzae*), have been identified as the established pathogens of rice (51). The current study reveals that the reduced occurrence of unclassified *Pleosporales* and *Cochliobolus* in conventional seeds, compared to organic seeds, may be attributed to the application of fungicides such as azoxystrobin and propiconazole in conventional rice cultivation. These fungicides likely inhibited the presence of these fungi. However, ITS does not provide full resolution for definitive species identification. Furthermore, *Auriculibuller* spp. were also observed in rice seeds grown in the organic farming systems, which have been previously reported as a member of aerial microbiota observed in rice fields (52). The usage of additional locus or metagenomic surveys in future studies can provide a more in-depth understanding of the diversity and functional implication of seed endophytic communities in rice grown under conventional and organic farming systems. When studying the plant endosphere, the host genome overwhelms the genetic composition within the total metagenomes. To gain deeper insights into seed communities, new techniques such as the “microbial bait” approach can be employed to capture and analyze the microbiota.

In our microbial survey study using culture-dependent methods, we found no difference in endophytic bacteria between rice plants grown under conventional and organic management systems. All the isolated seed endophytic bacteria were classified into 10 different genera. Notably, the bacterial groups *Panotea, Pseudomonas,* and *Xanthomonas* were isolated at a higher percentage, consistent with previously observed endophytic bacterial groups found in rice seeds (53–56). In this study, we identified other endophytic bacterial genera, including *Arthrobacter, Bacillus, Curtobacterium, Chryseobacterium, Paenibacillus,* and *Sphingomonas.* These genera have also been previously isolated as rice endophytic bacteria (54, 55). Many of these bacterial genera isolated in this study are known for forming beneficial symbiotic associations with plants, exhibiting various activities that promote plant growth and providing protection against phytopathogens (46, 53, 56). We evaluated the ability of these seed endophytic bacterial isolates to protect rice seed from the rice seedling blight pathogens. Our study demonstrated that two strains, one belonging to *Bacillus* spp. and the other to *Pantoea* spp., exhibited *in vitro* suppression of the three rice seedling blight pathogens, *R. solani* AG4, *R. solani* AG11, and *M. graminum*. Both *Bacillus* spp. and *Pantoea* spp. are well known for their pathogen-suppressing abilities (25, 57–61). *Burkholderia* spp. isolates were observed to enhance the radial growth of *R. solani* AG4. Bacterial-fungal symbiosis plays an important role in mutualistic growth and improved fitness of both bacteria and fungi (62, 63), as has been reported previously in both *Rhizoctonia* (64) and *Burkholderia* (63). However, further research is required to gain a better understanding of the ability of these bacterial isolates to protect rice seeds against seedling blight pathogens, as well as the potential role of these fungal symbionts in disease development and pathogenesis. Furthermore, these seed endophytic bacteria may possess several plant growth-promoting activities. However, the characterization of other plant growth-promoting activities exhibited by all bacterial genera isolated in this study is beyond the scope of our study.

The present study represents a proof of concept with limitations in terms of temporal sampling (single time point) and spatial extent (local) for evaluating the differences in microbial diversity within seeds between conventional and organic farming practices. Further studies are needed to establish a more comprehensive framework that can provide a deeper understanding of seed endophytes and their associations with farming practice. We also underline the necessity of conducting long-term studies that encompass diverse ecosystems relevant to rice agriculture. These studies should incorporate different management systems and geographic scales to gain a better understanding of the complexities of the farming systems and seed microbiota.

Additionally, we acknowledge the limitations of the culture-dependent approach employed in this study. Regardless, our study has provided valuable insights into the potential of these seed endophytic microbial populations. This highlights the significance of culture-based studies in assessing the functional capabilities of the observed microbiome differences. Additionally, the lack of taxonomic resolution in our culture-dependent approach could be attributed to the use of limited culture conditions. Emerging high-throughput culturomics methods, despite being costly, hold promise for future investigations, offering a more comprehensive understanding of the functional potential of seed endophytes, especially in relation to farming practices. These microbial populations have potential applications in modern agriculture, where scientists and growers constantly strive to adapt to changing climatic conditions, diminishing effectiveness of chemical-based protections, and evolving dynamics of phytopathogens.

In summary, we, for the first time, identified and compared the bacterial and fungal microbial communities within rice seeds produced under conventional and organic farming systems. Our results reveal significant differences in the seed microbial populations between both farming practices. Specifically, the bacterial endophyte populations in conventional rice seeds exhibited higher diversity compared to those in organic rice seeds, while the fungal endophyte populations showed greater diversity in organic rice seeds compared to conventional rice seeds. Additionally, we identified bacterial endophytes with potential as biological control agents against three seedling blight pathogens. Overall, our results contribute new insights into the structure and composition of seed endophytic microbial communities, as well as the influence of farming practice (conventional vs organic) on these communities. This understanding can help develop novel microbiome-based approaches, such as the creation of biotic stress-tolerant crops, to enhance plant health and improve crop productivity.

## MATERIALS AND METHODS

### Collection of seed samples

In this study, rice seed samples were collected in 2020 from two separate rice field trials located in half a kilometer apart at the Texas A&M AgriLife Research Center, Beaumont, Texas. Both fields possessed the same type of soil, classified as league type, with the following composition: 3% sand, 32% silt, 64% clay, 4% organic matter, and pH 5.5 (65, 66). One field was under conventional management (30^o^03′37.9″ N 94^o^17′34.7″ W), while the other followed organic practices (30^o^03′55.2″ N 94^o^17′51″ W). During the 2020 cropping season, both conventional and organic trials were conducted using a randomized complete block design with four replications. The plots were 4.9 m long and 1.3 m wide, consisting of eight rows, spaced 18 cm apart. Both field trials were drill seeded with the same rice cultivar Presidio at 134 kg/ha on 27 April. The seeds used for the organic rice trial were obtained from previous year’s organic crop. The conventional and organic trials were managed following local recommendations for conventional and organic rice production recommendations, respectively, regarding fertility, irrigation, pest control, and other agronomical practices (21, 33).

The conventional field had been cropped to conventional rice every other year for more than 20 years. A typical growing season for the conventional crop consisted of one or two applications of fungicides (azoxystrobin, a QoI complex 3 respiration inhibitor fungicide, 280 g a.i./ha and/or propiconazole, a sterol biosynthesis inhibitor fungicide, 319 g a.i./ha), one application of an insecticide (zeta-cypermethrin, a group 3A insecticide, 28 g a.i./ha), and three applications of herbicides (clomazone, 460 g a.i./ha; halosulfuron, 67 g a.i./ha; and penoxsulam, 40 g a.i./ha). Synthetic fertilizers, mostly urea (46-0-0, N-P-K), were applied at a total rate of 202 kg/ha to the field in each crop season. In the 2020 trial, plots received 56 kg N/ha of urea on 15 May and 168 kg N/ha of the fertilizer on 7 June. Permanent flood was established on 7 June. Plots were treated with the herbicides Command (clomazone, 0.94 L/ha) and Halomax (halosulfuron, 0.09 L/ha) on 25 April for weed control.

On the other hand, the organic field followed organic management practices and had been certified for organic rice production since 2007. No synthetic chemicals such as fungicides, insecticides, herbicides, and fertilizers (urea) were used. Instead, the organic-certified soil amendments Nature Safe (13-0-0, N-P-K), Rhizogen (7-2-1), and AgriRecycle (4-2-3) were utilized, along with winter cover crops such as white clover, purple clover, or annual ryegrass to provide nutrients for the organic rice crops. In the 2020 trial, plots received 56 kg N/ha of the organic-certified fertilizer Nature Safe on 14 May, 168 kg N/ha on 1 June, and 168 kg N/ha on 1 July. Permanent flood was established on 1 June.

Neither the conventional nor the organic trials receive fungicides or insecticides during the 2020 cropping season. At maturity, the plots in each trial were harvested using a plot combine. From each of the four plots in both trials, three subsamples of 0.9 kg seeds each were collected, resulting in a total of 12 seed samples per trial. All these seed samples were stored at 4°C until use.

### DNA extraction, library preparation, and sequencing

All seed samples were washed with 1% Tween 20 for 30 s before surface sterilization. The sterilization process involved using 10% bleach for 5 min, followed by 70% ethanol for 30 s. The seeds were immediately washed with sterile distilled water thrice and then stored at −20°C until processing. DNA from approximately 1 g of surface-sterilized seeds was isolated using DNeasy Plant Mini Kit (Qiagen, Valencia, CA, USA) with necessary modifications. This involved homogenizing five seeds per lysing matrix A (MPbio, OH, USA) using a Bead mill 24 Homogenizer (Thermo Fisher Scientific, CA, USA) at a speed of 4.0 m/s for 30 s, and the rest of the protocol was followed based on the manufacturer’s recommendations. DNA integrity was evaluated by electrophoresis on a 1% agarose gel, quality assessed in Quickdrop Micro-volume spectrophotometer (Molecular Devices, San Jose, CA, USA), and quantified using a Qubit Double-Stranded DNA High-Sensitivity Assay Kit (Thermo Fisher Scientific, MA, USA) in a Qubit 2.0 fluorometer (Invitrogen, CA, USA). Amplicon libraries were prepared by the Genomics and Bioinformatics Service at Texas A&M (College Station, TX, USA) using the primers 515F- GTG CCA GCM GCC GCG GTAA and 806R- GGA CTA CHV GGG TWT CTA AT (67) targeting the V4 region of 16S rRNA. Similarly, ITS-region-based amplicon libraries were prepared using primers ITS1F- CTT GGT CAT TTA GAG GAA GTAA and ITS2-GCT GCG TTC TTC ATC GAT GC (68). Libraries were sequenced on an Illumina Miseq PE250 platform by the Genomics and Bioinformatics Service at Texas A&M University (College Station, Texas, USA). Raw sequences have been deposited in NCBI Sequence Read Archive under Bioproject: PRJNA866104 and PRJNA866106 for 16S and ITS amplicon, respectively.

### Sequence processing

All the raw reads were processed using the Mothur v.1.47 (69). The protocols for the Mothur were applied following Miseq sop (https://mothur.org/wiki/miseq_sop/) with few modifications as needed. The total number of reads were 1,474,695 for 16S V4 amplicon sequences and 1, 132,133 sequences for ITS-based amplicon after quality filtering. Sequence reads exceeding 400 bp were considered too long for the 16S V4 analysis, while reads below 450 bp were considered too short for the ITS region analysis. In addition, any ambiguous bases and maximum repeat of a nucleotide sequence more than eight were removed using Mothur built-in function “maxambig” and “maxhomop.” One sample from the 16S amplicon was excluded from the analysis due to its poor total read quality, resulting in a total of 11 samples available for processing 16S V4 reads. Mothur built-in function was applied to make contigs, quality control of bad reads, removal of chimeras, and finally assigning operational taxonomic units. A 97% cutoff was applied to bacterial taxa, while a 95% cutoff was used for fungal taxa. Ribosomal Database project classifier (70) with the Silva database v138 (71) was used for taxonomy assignment of 16S rRNA reads. Similarly, Mothur release of UNITE database (72) was used for the taxonomic assignments of ITS reads. OTUs assigned to mitochondria, chloroplast, eukaryotes, or unknown were removed from 16S rRNA data sets, and OTUs were assigned. Similarly, unknowns were removed from ITS data sets. Before downstream analysis, any single-digit OTUs (<10) were removed from the data set to avoid spurious sequences (73).

### Diversity estimation and statistical analysis

Relative abundance data from both 16S rRNA and ITS data sets were analyzed in R-studio v2022.2.3.492 (74) with R version 4.1.2 (75) using Phyloseq v1.38 (76), Vegan v2.5.7 (77), ggplot2 (78), and tidyverse (79). Relative abundance bar graph was generated for both 16S rRNA and ITS data sets, and all taxa with <2% relative abundance were categorized in the “other” category. The alpha diversity measures were generated for observed, Shannon diversity, Simpson, chao1, and ACE in Phyloseq v1.38 (76). The Chao1 and ACE diversity indices were utilized to assess community richness, whereas the Shannon and Simpson diversity indices were employed to evaluate both richness and evenness within the community (80). Mann-Whitney *U* test of significance was conducted for all alpha diversity measures using “wilcox.test()” built-in function in R. PERMANOVA was calculated using “adonis()” function in Vegan v2.5.7 (77) to determine the effects of the two farming systems on the bacterial and fungal communities, with Bray-Curtis dissimilarity matrix on both 16S rRNA and ITS data set. NMDS plot was constructed based on the Bray-Curtis dissimilarity matrix for visualization. Differentially abundant OTUs were determined in both 16S rRNA and ITS data sets by “lefse” function (81), and indicator OTUs were determined using “indicator” function in Mothur v.1.47 (65). Indicator function in Mothur was adapted based on the Dufrene and Legendre (82), where they defined species to unique habitats independence of relative abundances. Default parameters were used for both “lefse” and “indicator.”

### Isolation and identification of culturable bacterial endophytes

Approximately 20 g of rice seeds was surface sterilized as described above and completely crushed in sterile mortar and pestle with phosphate-buffered saline (PBS) buffer. Crushed rice seeds were then transferred to sterile flask with 200 mL of PBS buffer to create a suspension solution. A serial dilution (100 µL) of the homogenized seed suspension was prepared in PBS buffer and plated on to tryptic soy agar (TSA) plates to determine the culturable population of seed endophytic bacteria. Serial dilutions of 10^−5^ and 10^−6^ fold were made, and 100 µL aliquots of each dilution were spread on separate TSA plates. Colony PCR was conducted to amplify 16S rRNA genes using the universal primers 27F: 5′- AGA GTT TGA TCC TGG CTC AG-3′ and 1492R: 5′-GTT TAC CTT GTT ACG ACT T-3′ (83). Amplified PCR products were purified using Zymoclean Gel DNA Recovery Kits (Zymo Research, CA, USA) using manufacturer’s recommendations. All sequences were performed by Eton Bioscience (Eton Bioscience Inc., San Diego, CA. USA). Consensus sequences were constructed from the obtained 16S rRNA sequences, and identifications were done by Blast in EzTaxon server (https://www.ezbiocloud.net/). The nucleotide sequences of 16S rRNA genes were deposited to the GenBank under the accession numbers: OP132284–OP132369.

### *In vitro* bacterial endophyte antagonism assay

Antagonistic activities of seed endophytic bacterial isolates were tested on the three rice seedling blight pathogens: *R. solani* AG4 (36), *R. solani* AG11 (38), and *M. graminum* (37). The isolates were initially dereplicated based on their 16S rRNA gene sequence. Those strains with 97% similarity were grouped together (84). One representative isolate was selected from each group for the *in vitro* assay.

The *in vitro* experiments were conducted using the dual culture method (58). All bacterial isolates were grown in LB broth (Thermo Fisher Scientific, Waltham, MA, USA) on an orbital shaker at 150 rpm at 28°C for 48 h. The bacterial suspensions were centrifuged at 4,032 g for 10 min in 50 mL sterile falcon tubes. The pellets were resuspended with sterile distilled water, and the final concentration of all bacterial isolates was adjusted to an OD of 0.3 at 600 nm for each isolate. For all fungal pathogen cultures, a 5-mm agar plug was taken from the edge of the colony of actively growing fungal cultures on Potato Dextrose Agar (PDA) plates and placed at the center of another PDA plate. Each of the four locations, equally spaced at a distance of 2 cm from the central fungal culture disk, received a drop (10 µL) of bacterial suspension. Sterile distilled water was used as the negative control. The test plates were incubated at 27°C. Each treatment was replicated three times, and the experiments were repeated twice. The mycelia growth (cm) of the three fungal pathogens, *R. solani* AG4, *R. solani* AG11, and *M. graminum*, was measured at different time points: 2, 3, and 5 days post incubation, respectively, taking into account distinct growth rates.

### Phylogenetic analyses

Phylogenetic analyses were performed using consensus 16S rRNA sequences of endophytic bacterial isolates that exhibited antagonistic activity against rice seedling blight pathogens. For each of the bacterial isolates, top hits in the databases were compared to generate a phylogenetic tree. All sequences were aligned with Silva database v138 (67), and alignment was edited and adjusted using Mothur v1.47 (65). On all the aligned sequences, neighbor-joining (85) phylogenetic analyses were performed in R-studio with R version 4.1.2 using APE package version 5.4–1(86). All trees were visualized and manually edited using FigTree v1.4.4 (http://tree.bio.ed.ac.uk/software/figtree/) (87).

## SUPPLEMENTAL MATERIAL

Supplemental tables and figures spectrum.03662-23-s0001.pdf Tables S1 and S2; Fig. S1 and S2.

ASM does not own the copyrights to Supplemental Material that may be linked to, or accessed through, an article. The authors have granted ASM a non-exclusive, world-wide license to publish the Supplemental Material files. Please contact the corresponding author directly for reuse.
