# Supplementary material for: Characterization of differences in seed endophytic microbiome in conventional and organic rice by amplicon-based sequencing and culturing methods
Source: Microbiol Spectr. 2024 Aug 13;12(10):e03662-23. doi: 10.1128/spectrum.03662-23 (PMC11448069; doi:10.1128/spectrum.03662-23)
Supplement: Supplemental tables and figures — Tables S1 and S2; Fig. S1 and S2. [file spectrum.03662-23-s0001.pdf]

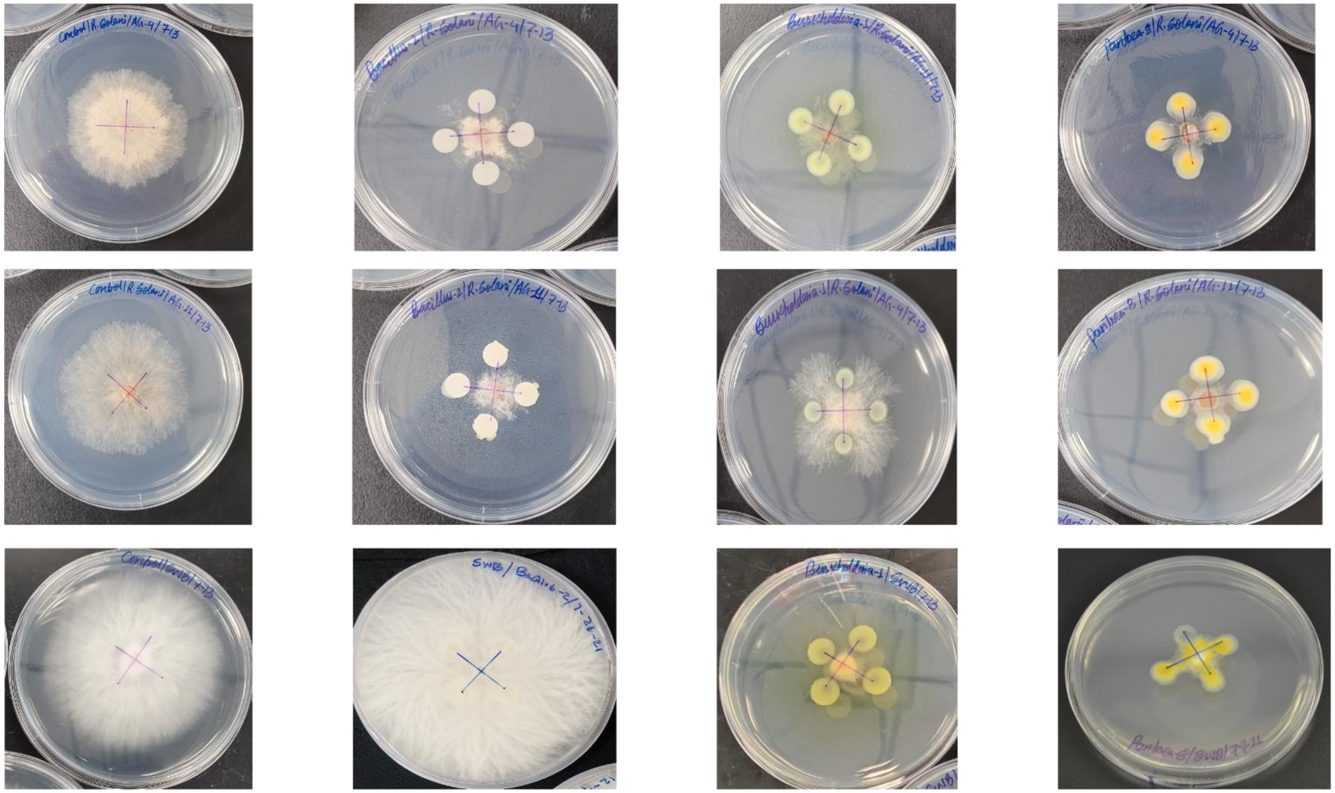

Supplementary FIG. 1. *In-vitro* antagonistic effect of three seed endophytic bacteria *Bacillus* sp. ST24, *Burkholderia,* sp. OR5, and *Pantoe asp*. ST25 against rice seedling pathogens. *R. solani* AG4, *R. solani* AG11, and *M. graminum.*

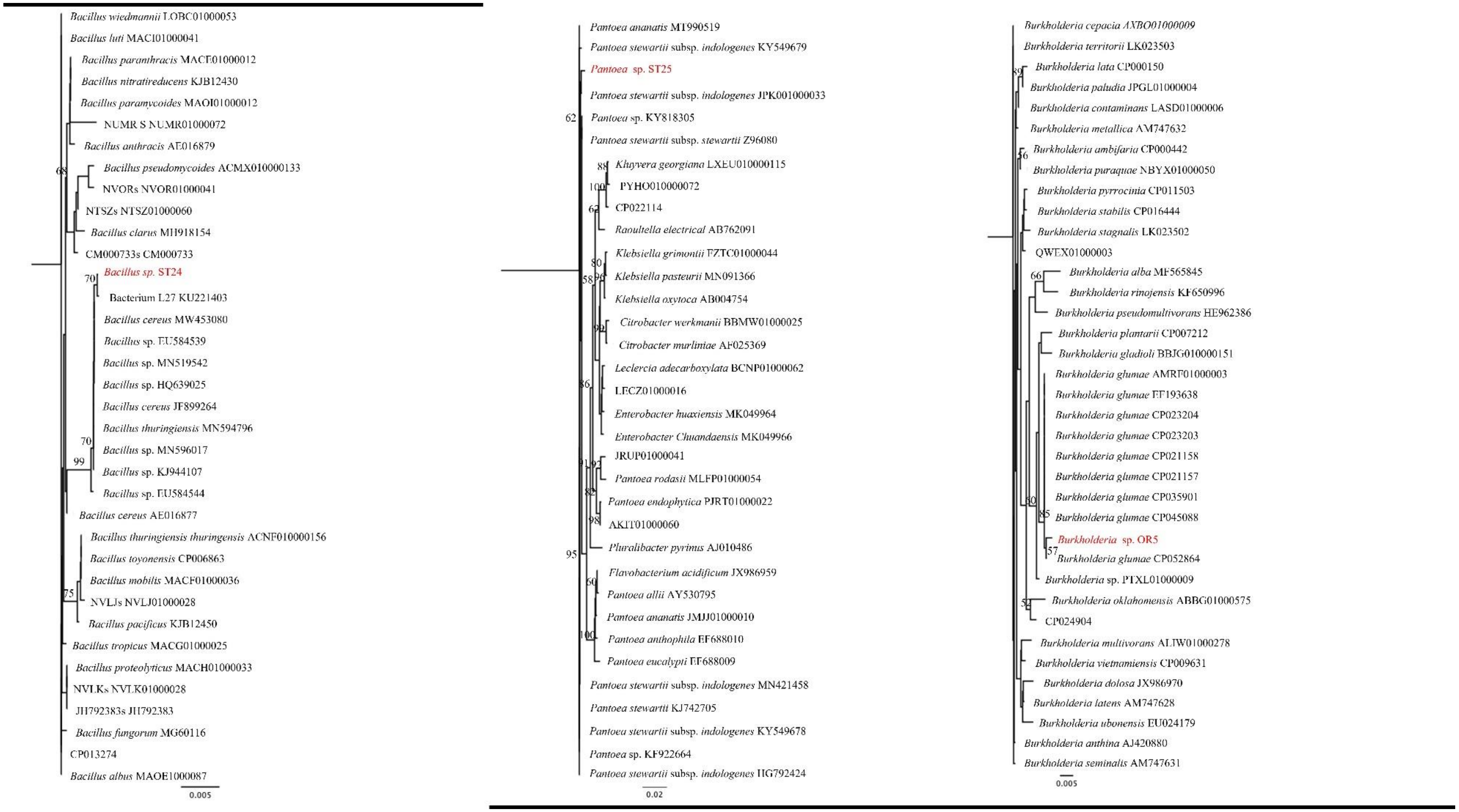

Supplementary FIG. 2. Neighbor-joining phylogenetic analyses of seed endophytic bacteria *Bacillus* sp. ST24, *Burkholderia* sp. OR5, and *Panotea* sp. ST25 with various *Bacillus, Burkholderia,* and *Pantoea* species from the databases.

Supplementary Table 1. Distribution of bacterial orders within the core community across all seed samples.

Sample ID	Taxon									
	Burkholderia	*Enterobacterales*	*Kineosporiales*	*Micrococcales*	*Paenibacillales*	*Pseudomonadales*	*Rhizobiales*	*Sphingomonadales*	*Xanthomonadales*	Other
OR1 Rep1	0.67	50.98	2.00	11.63	2.51	5.77	15.17	3.90	6.68	0.70
OR1 Rep2	0.64	53.68	2.21	12.20	2.14	5.45	12.17	3.78	6.95	0.80
OR1 Rep3	0.76	50.49	2.71	10.27	1.80	7.36	14.42	4.38	6.98	0.84
OR2 Rep1	0.44	51.52	2.36	10.27	2.59	6.33	14.65	4.42	6.60	0.82
OR2 Rep2	0.64	51.23	2.17	10.38	2.17	6.62	15.11	4.32	6.27	1.09
OR2 Rep3	0.51	54.17	2.20	8.62	1.90	6.70	14.15	4.63	6.34	0.78
OR3 Rep1	0.96	50.75	2.09	10.54	2.21	5.99	14.73	4.29	7.37	1.06
OR3 Rep2	0.76	50.96	2.24	10.19	2.15	6.04	16.08	4.56	6.31	0.70
OR3 Rep3	0.89	54.31	1.86	9.20	2.22	5.73	13.30	4.17	7.06	1.26
OR4 Rep1	0.54	52.49	2.03	11.00	2.05	6.68	13.65	4.61	6.17	0.77
OR4 Rep2	0.82	49.30	2.11	11.63	2.19	6.67	16.04	3.96	6.54	0.74
OR4 Rep3	0.77	57.29	1.72	10.32	2.02	6.28	11.48	4.00	5.85	0.26
ST1 Rep2	3.96	48.85	0.70	9.61	2.01	7.04	14.05	3.67	8.32	1.79
ST1 Rep3	4.18	47.64	0.91	8.51	2.53	7.44	14.77	3.32	8.82	1.88
ST2 Rep1	3.80	48.99	1.13	9.61	1.82	7.43	14.45	3.09	7.93	1.75

ST2 Rep2	3.32	50.37	1.09	8.70	2.18	6.77	15.00	3.06	7.96	1.55
ST2 Rep3	3.77	48.17	1.17	8.67	2.41	7.26	14.64	3.55	8.31	2.05
ST3 Rep1	3.35	51.09	0.97	8.81	2.09	6.63	14.61	3.46	7.76	1.22
ST3 Rep2	2.75	48.91	1.14	10.74	1.71	7.06	13.96	3.14	9.25	1.34
ST3 Rep3	2.80	49.66	1.13	9.46	2.12	6.52	14.37	3.69	8.82	1.43
ST4 Rep1	3.41	47.64	1.33	10.67	2.28	6.10	14.99	4.18	8.12	1.27
ST4 Rep2	3.60	46.51	1.24	9.93	2.28	7.22	15.64	3.66	8.29	1.61
ST4 Rep3	3.73	46.38	1.28	8.36	2.47	7.54	16.09	3.75	8.67	1.73

Supplementary Table 2. Distribution of fungal genera within the core community across all seed samples.

Sample ID	Taxon										
	Auriculibuller	*Cochliobolus*	*Phoma*	Unclassified	Unclassified	Unclassified	Unclassified	Other	Unclassified	Unclassified	Unclassified	Unclassified

				Mycosphaerellaceae	*Phaeosphaeriaceae*	*Pleosporaceae*	*Tremellales*		*Dothideomycetes*	*Phaeosphaeriaceae*	*Pleosporaceae*	*Pleosporales*
OR1 Rep1	2.64	11.22	8.80	0.35	10.67	2.63	0.00	7.21	0.04	3.24	11.37	41.83
OR1 Rep2	2.12	11.43	8.91	0.34	12.10	1.71	0.02	6.31	0.04	2.81	10.28	43.93
OR1 Rep3	1.77	13.55	7.05	0.46	10.92	1.92	0.00	6.28	0.05	2.85	11.83	43.33
OR2 Rep1	2.01	12.38	9.60	0.64	12.34	1.48	0.07	7.66	0.12	3.82	10.59	39.29
OR2 Rep2	2.63	11.73	8.76	0.43	10.79	2.70	0.00	6.78	0.03	3.32	10.92	41.89
OR2 Rep3	2.25	12.07	9.08	0.34	10.99	2.39	0.00	6.91	0.02	2.95	9.90	43.11
OR3 Rep1	2.42	13.04	9.19	0.16	11.47	2.23	0.00	7.00	0.00	3.19	12.97	38.34
OR3 Rep2	2.21	11.32	9.72	0.22	12.32	2.63	0.00	5.97	0.03	3.12	11.26	41.19
OR3 Rep3	2.64	4.36	14.35	0.58	15.92	1.68	0.00	5.84	0.00	2.32	8.21	44.10
OR4 Rep1	2.32	12.31	8.15	0.47	11.03	1.73	0.00	6.97	0.06	3.39	12.90	40.65
OR4 Rep2	1.72	11.83	9.05	0.11	13.19	1.95	0.00	6.27	0.00	2.83	11.09	41.96
OR4 Rep3	2.56	12.00	9.36	0.27	10.74	2.38	0.00	7.19	0.07	2.83	10.45	42.14
ST1 Rep1	0.09	2.63	53.54	1.90	11.07	1.78	1.17	2.22	2.14	0.06	2.26	21.14
ST1 Rep2	0.33	3.11	55.99	2.12	11.72	0.96	0.69	2.63	0.00	0.28	2.45	19.72
ST1 Rep3	0.24	2.77	49.81	1.58	13.44	1.44	1.53	2.55	1.56	0.21	2.31	22.56

ST2 Rep1	0.27	3.60	51.98	2.04	11.52	1.74	2.14	2.76	0.00	0.19	2.18	21.57
ST2 Rep2	0.18	3.35	53.53	2.26	12.44	1.39	1.11	2.72	0.01	0.24	2.14	20.64
ST2 Rep3	0.12	2.87	51.17	2.73	12.49	1.36	1.51	2.77	1.71	0.26	2.52	20.49
ST3 Rep1	0.39	3.41	53.43	2.05	11.56	1.39	1.10	2.23	1.29	0.18	1.89	21.08
ST3 Rep2	0.17	0.57	59.78	1.26	11.95	0.30	0.74	1.77	1.43	0.17	0.15	21.70
ST3 Rep3	0.49	3.20	52.78	2.12	11.75	1.27	1.24	2.61	0.00	0.22	2.29	22.04
ST4 Rep1	0.23	2.63	49.09	2.47	11.41	1.33	1.37	3.16	1.90	0.15	2.17	24.10
ST4 Rep2	0.50	3.35	52.70	1.97	12.04	1.29	1.04	2.34	2.21	0.40	1.66	20.51
ST4 Rep3	0.25	3.09	50.52	2.16	12.34	1.35	0.78	3.18	0.00	0.17	3.29	22.87
